# Negotiating ‘severity’ in plain sight

**DOI:** 10.1038/s41431-024-01685-w

**Published:** 2024-08-14

**Authors:** Aviad Raz

**Affiliations:** Dept. of Sociology & Anthropology, Ben-Gurion University of the Negev, Be’er Sheva, Israel

**Keywords:** Social sciences, Ethics

Germany has a unique legal framework where fetal anomaly itself is not an explicit legal ground for termination of pregnancy (TOP) or justification for prenatal screening programs. This contrasts with many other countries where severity of fetal conditions is a central criterion. Like much of German policy and legislation, dropping the former “embryopathic” (sometimes also called “eugenic”) indication by the German legislator in 1995 was driven, in part, by a desire to differentiate from historical, Nazi eugenic practices. It was meant as a declaration that life with or without disability deserves the same protection.

According to the German law from 1995, abortion is exempt from punishment if the conception is not older than 12 weeks, the abortion must be carried out by a doctor and the women must seek advice beforehand. A foetus’ genetically based disability can be interpreted as a threat to the physical or mental health of the mother and thereby serves as an indirect reason for a legal termination. This provides considerable elbow room, within a time- limit regulation (“Fristenregelung”), for decisions regarding “preventive abortions”. The paper by Nov-Klaiman, Bowman-Smart & Horn [1], in this issue, captures this paradoxical legal concealment of severity, the “elephant in the room,” with a very interesting and timely illustration of how both pregnant women and professionals still negotiate and are guided by perceptions of severity when making decisions around prenatal testing and TOP. I agree with the authors that the German case shows that removing criteria such as severity from legislation or policy is both futile and raises problems in practice. It is the absence of severity from genetic counseling that may reproduce ignorance and lead to decisions that are not well informed. This particularly concerns the recent increase in first trimester abortions in Germany that arguably aligns with the increase observed in NIPT uptake (now covered by public health insurance on a case-by-case basis). Indeed, critics have pointed out that NIPT has the potential (due to its use in early pregnancy) to raise new complexities in decision-making about abortion due to embryopathies. It therefore highlights the need for negotiating severity in plain sight so that parents can make well-informed decisions.

Explicitly acknowledging severity of fetal conditions as a legitimate factor impacting women’s wellbeing, while also emphasizing the socially contextualized and personal nature of severity, could better support autonomous reproductive decision-making in this legal environment. In 2012, the US National Down Syndrome Congress started a national campaign to ensure that pregnant women receive balanced information through a designated leaflet or (preferably) a representative of an organization for the relevant disability, at the time of prenatal genetic counseling, as mandated by the 2008 Kennedy-Brownback US legislation. We need balanced information on life with disability presented before/in genetic counseling, but it is unlikely that the re-introduction of “severity” by itself would rectify the situation.

The paradox of severity can be further highlighted by comparing the policy landscapes of prenatal and pre-implantation genetic testing. The German law is more protective concerning the extra-corporeal (IVF-PGD) embryo, whereas the intra-corporeal embryo/fetus is relatively much less protected [2]. The German embryo protection law (1991) includes, for example, forbidding HLA-PGD (for “savior siblings”) and stipulating that no more than three embryos can be created per cycle of IVF and they cannot be frozen or discarded. And yet, while the 1995 abortion law dropped the criterion of embryopathic severity, in 2011 negative selection via PGD against *severe* illnesses was allowed [3]. Why is severity used as a criterion in the context of pre-implantation genetic diagnosis (PGD) but not pre-natal diagnosis (PND)?

Indeed, a recent study [4] of public attitudes toward PGD in Germany shows how they diverge from the restrictive legislation, with widespread (secular) public support for allowing PGD not just for severe genetic diseases (as permitted by the new German law), but also for any genetic disease. This normative gap is unlikely to be resolved via electoral politics given the consensual, non-partisan approach to bioethical issues like PGD in Germany.

For health professionals, legal scholars, bioethicists, medical sociologists, and evidently all the people using these technologies, the existence of two distinct frameworks—one for the extra-corporeal embryo and one for the intracorporeal fetus—is intriguing. It may also hint at the positioning of women and their bodies in this debate. Severity itself, arguably, may be assessed in relation to cultural norms regarding gendered roles and expectations around motherhood and caring. This topic is, however, much too wide for a brief comment like this. It is also intriguing that the legal acknowledgement of severity as a criterion for selective prevention takes place in the extra-corporeal context of pre-implantation genetic diagnosis (PGD) – extra-corporality being defined in relation to the woman’s body. Allowing negative selection against *severe* illnesses via PGD, but not PND, reflects moral gradualism in practice.

With the advent of new genetic technologies, preimplantation screening is becoming a reality. TOP regulation, evidently, cannot be equally applied to a pre-implanted, fertilized egg in a glass tube. The difference may reflect a gradualist approach to the moral status at the beginning of life, which is not an all-or-nothing construction. Rather, as the fertilized egg is successfully implanted, and as the fetus matures, its interests and moral rights—including, most importantly, its rights as compared to the pregnant woman—strengthen. But then again, this highlights the question of why the extra-corporeal, “pre-” embryo should be given *extra* protection by the legislator, and what kind of protection is intended. As new scientific and technological developments emerge, Nov-Klaiman et al.’s [1] paper will provide an important frame for considering what new regulatory developments should be also developed.
